# Exposure to genetically engineered olive fly (*Bactrocera oleae*) has no negative impact on three non-target organisms

**DOI:** 10.1038/s41598-017-11908-4

**Published:** 2017-09-13

**Authors:** Thea Marubbi, Clare Cassidy, Esther Miller, Martha Koukidou, Enca Martin-Rendon, Simon Warner, Augusto Loni, Camilla Beech

**Affiliations:** 1 Oxitec Ltd., 71 Innovation Drive, Abingdon, Oxfordshire OX14 4RX United Kingdom; 2 Department of Agriculture, Food and Environment, University of Pisa, Via del Borghetto 80, 56124 Pisa, Italy; 3 Present Address: Unilever, 3 St. James Rd, Kingston upon Thames, KT1 2BA UK; 4 Cambea Consulting Ltd., 10 Beech Court, Wokingham Road, Hurst, Berkshire RG10 0RQ UK

## Abstract

*Bactrocera oleae* (Diptera: Tephritidae) remains a major pest of olive fruit production worldwide. Current pest management programs largely depend on chemical insecticides, resulting in high economic and environmental costs. Alternative pest control approaches are therefore highly desirable. We have created a conditional female-specific self-limiting strain of *B*. *oleae* (OX3097D-Bol) that could be applied for sustainable pest control. OX3097D-Bol olive fly carries a fluorescent marker (DsRed2) for identification and a self-limiting genetic trait that is repressed by tetracycline. In the absence of tetracycline, the tetracycline transactivator (tTAV) accumulates, resulting in female death at larvae and early pupal stages. The aim of this study was to evaluate the impact of genetically engineered OX3097D-Bol olive fly on three non-target organisms that either predate or parasitize olive flies, one from the guild of parasitoids (*Psyttalia concolor*) and two from the guild of predators (*Pardosa* spider species and the rove beetle *Aleochara bilineata*). No significant negative effect was observed on life history parameters, mortality and reproductive capacity of the non-target organisms studied. These results suggest that potential exposure to DsRed2 and tTAV gene products (e.g. mRNA and encoded proteins) would have a negligible impact on on-target organisms in the guilds or predators and parasitoids.

## Introduction

The olive fruit fly, *Bactrocera oleae* (Rossi) (Diptera: Tephritidae), is a major agricultural pest of olive fruit production across the world. The female olive fly lays eggs inside the olive fruit and larvae feed on fruit flesh causing extensive crop damage^1^. Current pest control programs rely heavily on chemical insecticides, which not only have high economic and environmental costs, but have resulted in the appearance of insecticide-resistant pest populations^2^. In addition, these chemistries not only act on pests but also affect beneficial insects. Thus, integrated pest management strategies that allow the use of control measures and have no adverse effect on beneficial insects offer further advantages to growers. Alternative pest control methods, such as the sterile insect technique (SIT), have been previously described for olive fly^3^. However, these have consistently yielded poor results, most likely due to the fitness penalties acquired by the radiation-sterilized flies^3–6^. SIT male-only releases are preferable and have been proposed as a new approach to improve pest control methods^4, 5^. Releases of males are cost-effective and ensure the reduction of the next generation’s population since they mate with wild females.

To address this, we have generated a conditional female-specific self-limiting strain (OX3097D-Bol) of the olive fly *B*. *oleae* that could be used in pest control programs. The genetically engineered OX3097D-Bol strain carries a fluorescent protein marker gene (DsRed2) that facilitates quick identification of all genetically engineered individuals at all life-stages. OX3097D-Bol also carries a conditional female-specific self-limiting gene that is dominantly inherited^7^. The conditional female-specific self-limiting gene is repressed by tetracycline, present in the larval dietary medium^8^. In the presence of tetracycline, the system is inactivated and both females and males can be reared in the laboratory. When tetracycline is absent from the rearing process, the tetracycline transactivator (tTAV) is accumulated in females leading to male-only survival^7^. Data from laboratory and greenhouse studies demonstrated that the release of OX3097D-Bol males could provide sustainable control of pest olive fly as they will mate with wild females and produce progeny that will die at larval and pupal stages^7^.

The assessment of the environmental impact of genetically engineered insects is an important safety aspect and a prerequisite for their commercialization as vector/pest control agents^9^. Risk of exposure to non-target organisms could occur through parasitizing the *B*. *oleae* larvae or direct feeding on *B*. *oleae* larvae or eggs^10^. Our study has tested the potential impact of genetically engineered olive fly *B*. *oleae* on three relevant non-target organisms, one from the guild of parasitoids (*Psyttalia concolor* (Szépligeti), Hymenoptera: Braconidae) and two from the guild of predators (*Pardosa* spiders (Koch) (Araneae, Lycosidae) and the rove beetle *Aleochara bilineata* (Gyllenhal) (Choleoptera: Staphylinidae)).

*P*. *concolor* is a larval-pupal endoparasitoid that develops within a range of Tephritid larvae including medfly (*Ceritatis capitate* (Wiedemann) (Diptera: Tephritidae)) and olive fly (*B*. *oleae*)^11^. *P*. *concolor* has a global distribution spanning Africa, Europe, America, Central America and Pakistan as a result of accidental or intentional introduction for use as a biological control agent^12, 13^. Female *P*. *concolor* detect olive fly larvae by the vibrations made when burrowing through the fruit. The female parasitoid wasp lays an egg inside the larvae, through the fruit, by using her ovipositor^12^. Once the parasitoid egg is within its host, the host larvae continues to feed on the fruit until it is ready to pupate. Whilst the host is pupating, the parasitoid egg hatches and the parasitoid larvae feeds on the host, causing its death.

*Pardosa* species (wolf spiders) are generalist predators, attacking a range of live prey species, including Diptera. They can be found in large numbers in agricultural fields in the spring and summer months. Spiders from the Pardosa genus have been identified in citrus orchard in Spain^14, 15^ and in olive groves in the Mediterranean^16^. *Pardosa* species are often the organism of choice for testing the effect of plant protection products on non-target organisms^17^. In such experiments, *Pardosa* are caught from the wild as there is no reliable laboratory breeding protocol^17^. Mature females and males mate in the early spring and females can be seen carrying an egg sac from May onwards.

Species of the genus *Aleochara* (Coleoptera: Staphylinidae) are predators of olive fly. *A*. *bilineata* is normally reared of the pupae of cyclorrhaphous Diptera. First instar larvae enter the host puparium, and after having consumed the host and completed the three larval stages, they pupate inside the host puparium. Beetles then emerge from the host pupae. In this study, pupae of the onion fly Delia antiqua (Meigen) (Diptera: Anthomyiidae) were used as host organism for the larvae of *A*. *bilineata*. Reproductive success of beetles is usually measured by counting the number of second generation beetles (offspring) emerging from the onion fly pupae.

Here we assessed the effect of the exposure to genetically engineered OX3097D-Bol olive fly, compared to wild-type, on: (i) pupal emergence rate of the olive fly parasitized by *P*. *concolor* and life history parameters of the parasitoid, (ii) mortality and behavior of *Pardosa* spiders when fed olive fly larvae and (iii) mortality and reproductive success of *A*. *bilineata* fed olive fly larvae.

## Results

The development of the genetically engineered OX3097D-Bol olive fly strain tested in this study has been described elsewhere^7^. OX3097D-Bol contains a fluorescent protein marker gene (DsRed2) and a conditional female-specific self-limiting genetic trait which in the absence of tetracycline allows the accumulation of the tetracycline transactivator (tTAV) gene products resulting in female death at larval/pupal stages^7^. There are currently no known methods for accurately sexing olive fly larvae whilst alive. Therefore, in this study we used mixed sex OX3097D-Bol and wild-type larvae reared on tetracycline. To expose the non-target organisms to both DsRed2 and tTAV gene products (e.g. mRNA and encoded proteins) olive fly larvae or adults were then fed fresh diet without tetracycline.

### Wild-type and OX3097D-Bol olive fly are equally adequate hosts of the parasitoid *P. concolor*

**Figure 1:**
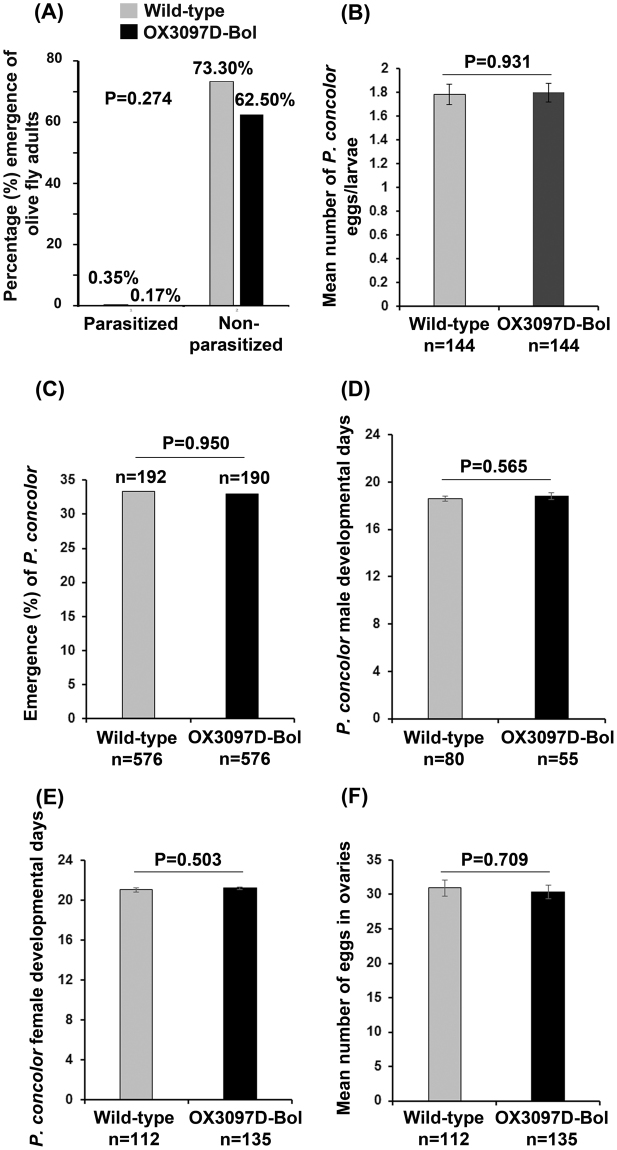
Life history parameters for *Psyttalia concolor* reared on either wild-type or OX3097D-Bol *Bactrocera oleae* larvae. Wild-type (n = 720) and OX3097D-Bol (n = 720) *B*. *oleae* (olive fly) third instar larvae reared in the presence of 100 μg/mL of tetracycline were presented to *P*. *concolor* naïve females (n = 240) at a 1:3 ratio (parasitoid female: olive fly larvae) for 30 min. Approximately 20% of the parasitized olive fly larvae (n = 144) were dissected to determine the level of parasitism. The remaining 80% of parasitized olive fly larvae (n = 576) were transferred to Petri dishes of fresh diet without tetracycline and allowed to emerge. (**A**) Percentage emergence of adult olive fly parasitized by *P*. *concolor*. Emergence from parasitized olive fly was estimated to be 0.35% and 0.17% for wild-type and OX3097D-Bol, respectively. Emergence from non-parasitized control olive fly was 73.3% for wild-type and 62.5% for OX3097D-Bol. (**B**) Mean number of *P*. *concolor* eggs laid into olive fly larvae. (**C**) Percentage of emergence of adult *P*. *concolor* from the parasitized olive fly larvae. (**D**) Mean number of days from parasitism to the emergence of adult male wasps (developmental days). (**E**) Mean number of days from parasitism to the emergence of adult female wasps (developmental days). (**F**) Mean number of eggs in *P*. *concolor* female ovaries. For non-proportional data, the numbers represent mean and standard error of the mean (±SEM). P-values ≤0.05 were considered statistically significant.

To determine whether exposure to OX3097D-Bol olive fly has any effect on the parasitoid *P*. *concolor*, female wasps were presented with third instar olive fly for oviposition. Predictably, olive fly larvae exposed to *P*. *concolor* did not survived parasitism. The percentage of parasitized olive fly adults that emerged was approximately 0.35% and 0.17% for wild-type and OX3097D-Bol larvae, respectively, in contrast to 73.33% and 62.50% emergence of non-parasitized wild-type and OX3097D-Bol larvae (Fig. 1A). The difference in emergence in non-parasitized and parasitized wild-type versus OX3097D-Bol olive fly larvae was not statistically significant (P = 0.274). There was no significant difference between the mean number of *P*. *concolor* eggs laid into OX3097D-Bol (n = 144) or into wild-type (n = 144) olive fly larvae (P = 0.931, Fig. 1B). There was also no significant difference between the percentage of *P*. *concolor* adults emerging from wild-type larvae (33.33%; n = 192 out of 576) and that emerging from OX3097D-Bol larvae (32.99%; n = 190 out of 576) (P = 0.95, Fig. 1C). Furthermore, no significant difference in the length *of P*. *concolor* developmental time between the treatments was observed when males (wild-type, n = 80 and OX3097D-Bol, n = 55; P = 0.565) and females (wild-type, n = 112 and OX3097D-Bol, n = 135; P = 0.503) were directly compared to each other (Fig. 1D and E).

The reproductive potential of newly eclosed *P*. *concolor* females, as determined by the number of fully developed eggs in both ovaries, did not vary significantly (P = 0.709) between females that parasitized wild-type larvae (n = 112) and those that parasitized OX3097D-Bol larvae (n = 135) (Fig. 1F).

Taken together, these data suggest that both olive fly strains, wild-type and OX3097D-Bol, were perceived as equally adequate hosts by *P*. *concolor* females.

### Feeding *Pardosa* spiders with OX3097D-Bol has no detrimental effects

To assess the effect of a diet consisting of genetically engineered compared to wild-type olive fly on *Pardosa* species food consumption, mortality and reproductive potential, spiders were fed exclusively on olive OX3097D-Bol or wild-type flies. Most spiders captured were females, each carrying an egg sac. Only 4/70 spiders captured were males, most likely because male spiders would have died, once mating has occurred early in the season^17–19^. The four male spiders collected were randomly allocated to the three study groups; 1 male was allocated to the OX3097D-Bol group (Treatment group), 1 male to the wild-type group (Control 1 group) and 2 males to the starved group (Control 2 group). The female spiders had their egg sac removed seven days before the start of the study (as experimental design in Supplementary Figure S1). All spiders were fed adult OX3097D-Bol or wild-type olive flies for 14 days.

Food consumption was consistently high over the duration of the study (Table 1), and there was no sign of avoidance or preference behavior towards either the wild-type (n = 30) or OX3097D-Bol (n = 30) olive flies. There were no significant differences in the mean number of flies consumed per spider per feeding session between the Treatment and Control 1 groups (see specific P-values in Table 1). Similarly, there was no significant difference (P = 0.841) in the total number of flies consumed by spiders fed with wild-type (total = 10.92 ± 1.83) or OX3097D-Bol (total = 10.97 ± 1.98) olive flies. There were five dead spiders in total by the end of the study, three of which were males (one from the Treatment group and two from the Control 2 group). There was no significant difference between mortality rates of spiders fed a diet consisting of OX3097D-Bol olive fly (n = 30) compared to those fed wild type olive fly (n = 30; P > 0.999) (Table 1). As expected, there was a significant difference between mortality rates of *Pardosa* spiders fed with OX3097D-Bol olive fly (n = 30) compared to starved spiders (n = 10; P = 0.019).

**Table 1:** Consumption of olive flies by *Pardosa* spiders and mortality rate of spiders fed a diet of olive flies.

Group (number of *Pardosa* spiders)	Diet supplied	Mean number of consumed flies per live spider per feeding ± SD*						Mortality rate (Number of dead spiders/total)
		Day 1	Day 4	Day 6	Day 8	Day 11	Day 13	
Treatment (n = 30)	OX3097D-Bol olive fly	1.95 ± 0.20	1.93 ± 0.26	1.95 ± 0.20	1.86 ± 0.42	1.93 ± 0.22	1.62 ± 0.63	0.03 (1/30)
Control 1 (n = 30)	Wild type olive fly	1.85 ± 0.31	1.85 ± 0.40	1.87 ± 0.43	1.90 ± 0.31	1.80 ± 0.50	1.62 ± 0.60	0.03 (1/30)**
Control 2 (n = 10)	Starved	NA	NA	NA	NA	NA	NA	0.30 (3/10)***
	P-value	0.383	0.41	0.638	0.695	0.422	0.601	

(*) Spiders were fed 2 flies per feeding session over a period of 14 days. Data analysis was conducted using the Wilcox rank sum test for non-parametric data. There were no significant difference in food consumption between spiders fed with OX3097D-Bol or wild-type olive flies per feeding session.

(**) Comparison between Treatment and Control 1 (two-tailed Student’s t-test). P ≤ 0.05 are considered statistically significant.

(***) Comparison between Treatment and Control 2 (two-tailed Student’s t-test). P ≤ 0.05 are considered statistically significant.

NA = not applicable; SD = standard deviation.

Although female spiders had their egg sac removed at the beginning of the study, a number of females (seven spiders from the Control 1 group and nine spiders from the Treatment group) had produced a new egg sac during the course of the study. None of the spiders in the starved group (Control 2 group) produced a new egg sac. This implies that both wild-type and OX3097D-Bol olive fly diets are equally adequate for female spiders to produce potential off-spring.

These data suggest that there is no significant difference in survival or behavior of *Pardosa* spiders when they are fed exclusively OX3097D-Bol flies or wild-type flies.

### Feeding rove beetles with OX3097D-Bol has not effect on their reproductive capacity

To assess the effect of a diet of genetically engineered olive fly on the full reproductive cycle of the predator *A*. *bilineata*, rove beetles were fed OX3097D-Bol or wild-type olive flies. Rove beetles were exposed to either genetically engineered OX3097D-Bol (Treatment group; n = 20) or wild-type (Control 1 group; n = 20) olive flies. As additional controls, beetles were also exposed to a diet of *Chironomus* species in the absence (Control 2 group; n = 20) and presence (Control 3 group; n = 20) of a toxic reference substance (ROGOR PLUS) known to reduce their reproductive capacity drastically at the dose used (440 g active substance/ha). An average of 450.3 beetles out of 6000 (approximately 26.7%) emerged from onion fly pupae when fed wild-type olive fly larvae (Control 1 group). In the toxic reference substance group (Control 3 group), the reproductive capacity of *A*. *bilineata* was reduced by >50%. Therefore, and according to the criteria selected *a priori* ^19^, the study was considered valid.

In this study, 28-day mortality data and cumulative fecundity data of adult rove beetles fed with a diet of OX3097D-Bol (Treatment group) or wild-type (Control 1group) olive fly larvae were compared. Mortality after 28 days was 6.25% in the Treatment group, 12.5% in the Control 1 group and 10.0% in the Control 2 group whilst it was 83.8% in the Control 3 group (Table 2). There was no significant difference between mortality in the Control 1 group (beetles fed with 0.10 g wild-type olive fly larvae) and the Treatment group (beetles fed with 0.10 g of OX3097D-Bol olive fly larvae) after 28 days (P = 0.222). By contrast, there was a significant difference between mortality in the Control 1 group and Control 3 group (P = 0.004).

**Table 2:** Mean mortality and fecundity of rove beetles *A*. *bilineata* fed a diet of olive flies.

Group	Rove beetle diet (larvae/test unit)	Mean mortality ± SD (%)	P- value^†^	Offspring/Beetle ± SD*	Fecundity effects**	P- value***
Treatment 1 (n = 20)	0.10 g OX3097D-Bol larvae	6.25 ± 9.46	0.222	40.30 ± 3.04	−0.47	0.079
Control 1 (n = 20)	0.10 g wild-type larvae	12.50 ± 6.45	—	40.50 ± 1.83	NA	—
Control 2 (n = 20)	0.10 g *Chironomus* larvae	10.00 ± 9.12	—	25.30 ± 3.62	NA	—
Control 3 (n = 20)	0.10 g *Chironomus* larvae + ROGOR PLUS (440 g a.s./ha)	83.80 ± 13.15	0.004	1.77 ± 0.60	−95.6	0.002

(†) Comparisons were made between Treatment and Control 1 or Control 3 and Control 1 (two tailed Student’s t-test). P ≤ 0.05 are considered statistically significant. (*)Mean number of offspring per beetle. (**) Fecundity effects: The relative reproductive performance will be expressed as percent reduction compared to the Control 1 group. (***) Comparisons were made between Treatment and Control 1 or Control 3 and Control 1 (two tailed Student’s t-test). P ≤ 0.05 are considered statistically significant. a.s. = active substance; NA = not applicable; SD = standard deviation.

Fecundity effects were estimated by comparing the mean number of offspring produced per beetle in the Treatment group with controls (Table 2). Fecundity in the Control 1 group was 40.5 offspring per beetle, whilst in the Control 2 group fecundity was 25.3 offspring per beetle. The reduction of the reproductive capacity in the Control 3 group (toxic reference substance) was −95.6%. There was no significant difference between the cumulative fecundity in the Treatment group and Control 1 group (P = 0.079). As expected, there was a significant difference in fecundity between the Control 1 group and the Control 3 group (P = 0.002). No other behavioural effects were observed with the test item treatment.

These results suggest that there is no significant difference in rove beetles’ life cycle when they are fed exclusively OX3097D-Bol flies or wild-type flies.

## Discussion

*B*. *oleae* is a major pest of olive fruit that is currently controlled through the application of chemical insecticides. However, it is acknowledged that the use of these insecticides poses several risks to pest control management. On one hand, these pesticides are suffering from loss of registration (e.g. the neonicotinoids in the European Union)^20^ and the development of resistance in the target pest insects (including the synthetic pyrethroids, carbamates)^21–24^. On the other hand, these chemistries have a broad spectrum and not only act on pest insects but also affect beneficial insects such as pollinators, predators and parasitoids. Therefore, and to offer further advantages to growers, it is crucial to develop integrated pest management strategies that are not harmful to beneficial insects.

Sterile insect Technique (SIT) is an alternative pest control method unsuccessfully trialed for olive fly^3^ and thus, male-only release has been proposed as a new approach to improve pest control programs^5^. We have generated a conditional female-specific self-limiting strain (OX3097D-Bol) of the olive fly *B*. *oleae* that could be used in pest control programs, as the female progeny do not survive to adulthood, in the absence of tetracycline. Release of self-limiting olive flies would be an effective and direct, species-specific pest management approach by preventing survival of female progeny. OX3097D-Bol males released into olive groves could subsequently mate with wild females. Those wild female flies that had mated with OX3097D-Bol males would produce progeny that would die at the larval/pupal stages. Exposure of parasitoids and predators to the transgenes and/or their products (e.g. mRNA and encoded proteins), could potentially have adverse effects on such non-target organisms. In this study, we have demonstrated for the first time that exposure (via diet or predation routes) to genetically engineered olive fly *B*. *oleae* has no detrimental impact on three non-target organisms, one from the guild of parasitoids (*P*. *concolor*) and two from the guild of predators (*Pardosa* spiders and rove beetle *A*. *bilineata*).

The Braconid wasp *P*. *concolor* was selected for this study as it can develop within a range of Tephritid larvae^11^ and because of its global distribution throughout four different continents^12, 13^. Mass rearing of *P*. *concolor* in laboratory conditions relies on a delicate balance of three factors: a) the parasitoid/host ratio, b) host exposure time and c) female status (naïve or experienced)^25^. Fruit fly larvae parasitized by *P*. *concolor* do not generally survive. It is possible for the host to encapsulate the parasitoid egg and survive to adulthood, however, the frequency of this occurrence for *P*. *concolor* parasitoids and their hosts is unknown^26^. In our study, most olive fly larvae (>99.5%), whether genetically engineered OX3097D-Bol or wild-type larvae, did not survive following exposure to *P*. *concolor*. In agreement with previous studies, there was also very low level of hyperparasitism or superparasitism^11^. Levels of *P*. *concolor* emergence >35% are rarely reported^13, 27^. A study using wild olive fly larvae, collected from the field and parasitized in the laboratory, reported 6.2% *P*. *concolor* emergence^28^, whilst 28.2% emergence has been observed from lab reared medfly^28^. In the present study, emergence of *P*. *concolor* from laboratory reared OX3097D-Bol and wild-type olive fly is close to the emergence level detected for laboratory reared medfly. No changes in life history parameters of *P*. *concolor* where observed when exposed to genetically engineered OX3097D-Bol olive fly compared to wild-type olive fly. In addition, exposure to genetically engineered OX3097D-Bol olive fly compared to wild-type does not affect the reproductive potential of *P*. *concolor* females.

*Pardosa* spiders captured in May were overwhelmingly females, carrying an egg sac. In the wild, the first spiders to die tend to be male, as their natural mortality radically increases after reproduction^19^. Given that most *Pardosa* females in our study were carrying an egg sac on capture, we can safely assume males have reached their reproductive phase. Removal of the first egg sac from the females appears to have advanced their development, as even though *Pardosa* are known to produce a second egg sac, this normally occurs from mid-July to September^17^. These results suggest that *Pardosa* spiders fed OX3097D-Bol olive flies could potentially produce as much offspring as those fed with wild-type olive flies. Wild caught *Pardosa* fed 100% OX3097D-Bol or wild-type flies did not show any avoidance behavior towards the genetically engineered olive fly compared to wild-type. This study falls within the limits of validity^19^, therefore, we accept the null hypothesis that there is no significant difference between the levels of mortality or behavior of spiders fed a diet consisting of genetically engineered OX3097D-Bol olive fly compared to those fed a diet consisting of wild-type olive fly. Consequently, we can conclude that DsRed2 and tTAV gene products are unlikely to be harmful to *Pardosa* spiders at 100% of diet. As *Pardosa* spiders are generalist predators and consume a wide variety of prey^29^, our results are likely to represent a significant safety margin.

Here, we have also assessed the effect of ingestion of *B*. *oleae* OX3097D-Bol on the mortality and reproductive capacity of *A*. *bilineata*. In the *Pardosa* studies, we concluded that female spiders fed genetically engineered OX3097D-Bol olive flies had similar reproductive potential as those fed wild-type olive flies. The study with *A*. *bilineata* is important as the test covered the entire life cycle of this predator and its overall reproductive capacity was used as endpoint. *A*. *bilineata* is a parasitoid of the pupae of cyclorraphous Diptera. Rove beetles fed with genetically engineered OX3097D-Bol olive fly did not show increased mortality or reduced reproductive capacity compared to those fed with wild-type olive fly or *Chironomus* species. No other effects were observed. Having assessed changes in the whole life cycle of this predator, we can conclude that any residues of DsRed2 and tTAV gene products from the genetically engineered OX3097D-Bol olive fly are unlikely to affect the reproductive capacity of *A*. *bilineata*.

Our results are in agreement with previously published data on safety of genetically engineered mosquitoes carrying similar transgenes and their exposure to non-target organisms^9^. Importantly, the DsRed2 marker gene and its protein have been widely used in a variety of species, including insects, without any adverse effect^30^. In mosquitoes, the tTAV expression system is repressed by tetracycline, but the corresponding mRNA has been shown to be expressed in the absence of the antibiotic^31^. In that previous study, no adverse effects were detected in *Toxorhynchites’* development fed with mosquito larvae reared either in the presence or absence of tetracycline^9^.

Non-target organisms such as parasitoids and predators may be exposed to the transgenes and their products directly from consuming insects that express and/or accumulate those products. The results presented here suggest that potential exposure to OX3097D-Bol olive flies carrying DsRed2 and tTAV genes, have no detectable impact on the lethal and sub-lethal life history traits of non-target organisms included in this study and by extrapolation, on other members of ecological guilds of predators and parasitoids as relevant sensitive indicator species were chosen for this study.

## Materials and Methods

### Olive fly strains

The *B*. *oleae* Argov strain was used as wild-type (kindly provided by the Israeli Agricultural Research Organisation (ARO) and Bio-Fly Ltd). *B*. *oleae* Dacus (Democritus laboratory, Greece) was used as a background strain to create the OX3097D-Bol strain, as previously described^7^. Briefly, OX3097D-Bol contains a fluorescent protein genetic marker (DsRed2), regulated by the immediate early gene 1 (*ie1*) promoter and homologous region 5 (Hr5) enhancer (Hr5/*ie1*) from the *Autographa californica* multicapsid nucleopolyhedrovirus (AcMNPV)^32–34^. OX3097D-Bol also contains a conditional female-specific tetracycline transactivator (tTAV) expression system linked to the fluorescent marker^8^. The sex-specific splicing module of the Mediterranean fruit fly *C*. *capitata* transformer (Cc*tra*) gene was linked to the tTAV gene coding region (Ant *et al*., 2012). The tTAV gene is under the control of a minimal promoter linked to the tetracycline responsive operator (TetO). In the absence of tetracycline, intracellular levels of tTAV gene products (e.g. mRNA and encoded proteins) accumulate in sufficient quantities that OX3097D-Bol females die at larval or early pupal stages. In the presence of tetracycline (100 μg/ml), tTAV expression is repressed allowing for normal female development^8, 31^.

### Olive fly rearing

Olive fly larvae (OX3097D-Bol and wild-type) were reared in 30mm Petri dishes on an artificial diet as previously described^7^. For the *Pardosa* and *P*. *concolor* experiments, olive fly larvae were reared in the presence of tetracycline (final concentration 100 μg/ml) added during the rearing process. For the *Pardosa* study, adults eclosed from larvae reared on tetracycline were then reared without tetracycline and therefore, spiders fed on OX3097D-Bol adult female olive flies should be exposed to tTAV gene products. Adult olive fly lines (OX3097D-Bol and wild-type) were housed in BugDorm cages (Model DP1000B) and reared as previously described^7^. OX3097D-Bol and wild-type olive flies were reared at 23 °C (±2 °C), 50% (±5%) relative humidity and 12 h:12 h Light/Dark cycle. Olive fly larvae for the *A*. *bilineata* study were reared without tetracycline.

### *P. concolor* rearing

*P*. *concolor* infested Mediterranean fruit fly (medfly) pupae were supplied by the Department of Agriculture, Food and Environment, University of Pisa, Italy. *P*. *concolor* adults emerged into a cage (length 50 cm, width 20 cm, height 25 cm) which housed around 300–400 individuals. Adult *P*. *concolor* were fed a honey (Rowse): pollen (100% pure Bee Pollen) mixture (1:1 ratio) and fresh water to drink, both *ad libitum*, and maintained in a room at 25 °C (±2 °C), uncontrolled humidity 35–50%, with a 16:8 light:dark cycle. Males *P*. *concolor*, emerged on average 2 days before females^27^. To ensure all females had equal opportunity to mate, female *P*. *concolor* were selected for experimental cages between 5–12 days following the first male emergence. Prior to the start of the experiment, all females were naïve (no previous experience of egg laying).

### *P. concolor* study

Each treatment cage (3.7 L, see Supplementary Figure S2) contained 30 *P*. *concolor* females and 10 *P*. *concolor* males. For each of the eight replicates the following cages were set up:

1. Treatment 1: *P*. *concolor* presented with 90 OX3097D-Bol third instar olive fly larvae
2. Treatment 2: *P*. *concolor* presented with 90 wild type third instar olive fly larvae
3. Control 1: 50 OX3097D-Bol third instar olive fly larvae
4. Control 2: 50 wild type third instar olive fly larvae

The control cages contained no parasitoid wasps. OX3097D-Bol and wild-type larvae were handled in the same manner and placed in a nylon exposure net bag which was wrapped around pipe insulation (as seen in Supplementary Figure S2) and hung into the cage without any parasitoids present. This provided a baseline for the survival of olive fly larvae after being handled via this method.

Third instar olive fly larvae (9–10 days old) were collected from artificial diet or sand and placed in a single layer inside a nylon fine mesh bag which was then wrapped around pipe insulation and secured in place by elastic bands (Supplementary Figure S2). Parasitoids were exposed to olive fly larvae for oviposition at a 1:3 ratio (parasitoid females: olive fly larvae) for 30 min, after which *P*. *concolor* females were encouraged to fly away from the nylon bag by lifting the tubing up a short distance and blowing them off the nylon mesh bag into the cage. The nylon mesh bag, with larvae inside, was then removed from the test cages and the hole was covered with a Petri dish lid.

Larvae were then tipped into a Petri dish where a representative subsample (approximately 20%) of the larvae from the test cages were collected. To determine the level of parasitism and check that the exposure period and larvae to parasitoid ratio, was appropriate, these larvae were dissected to count the number of parasitoid eggs within their bodies. The remaining larvae (approximately 80%) were placed onto a Petri dish of fresh diet in a plastic box with sand lining the bottom and allowed to feed or pupate. Pupae were collected 2 days later from the boxes and placed in Petri dishes until emergence. At 25 °C *P*. *concolor* were expected to emerge in 17–18 days^27^. Any pupae not emerged on day 27 post-larval exposure to parasitoids were considered dead.

Olive fly were allowed to emerge and die in the Petri dish and counted/sexed once all had eclosed. To record the developmental time of each eclosed parasitoid wasp, *P*. *concolor* were collected on the same day they eclosed (from day 17–27) post-parasitoid exposure and stored in 70% ethanol in a 1.5 ml microtube (Eppendorf) at room temperature. All newly eclosed females from each cage were dissected to identify the number of mature eggs in both ovaries.

### *Pardosa* spider collection and rearing

*Pardosa* spiders were collected in May 2014 from an allotment (compost heap, greenhouse, vegetable patch) in Faringdon (51^o^ 39′ 28″ N, 1^o^ 34′ 57″ W), Oxfordshire, UK. This study did not interfere with any endangered or protected species. Spiders were collected within a 10 m^2^ area on the same collection day and placed individually into large Drosophila vials (diameter x height = 2 × 6 cm) sealed with a ball of cotton wool. Attention was taken to avoid spiders losing limbs during the process. All captured *Pardosa* spiders had a mottled brown and black appearance with a beige stripe down their cephalothorax (top body segment) through the black coloration and the characteristic eye pattern of Lycosidae (wolf spiders). Spiders were caught 10 days prior to the start of the experiment as previously described^19^, acclimated to the laboratory conditions and deprived of food. Spiders were reared in a temperature controlled room at 23 °C (±1 °C), 50% humidity and 12 h:12 h light/dark cycle. Humidity within the vials was recorded to be around 70% using a digital recorder. Spiders were transferred to fresh vials with fresh water and sand weekly and they were fed once every two days on living OX3790D-Bol or wild-type adult olive flies.

### *Pardosa* study

The study was designed to include three groups:

1. Treatment: 30 *Pardosa* spiders fed OX3097D-Bol olive flies
2. Control 1: 30 *Pardosa* spiders fed wild-type olive flies
3. Control 2: 10 Starved *Pardosa* spiders

The weight of the spiders was measured prior to the start of the study and spiders were allocated a number so that they could be traced for the duration of the study. Spiders were ranked by their weight and randomly assigned to Treatment, Control 1 and Control 2 groups, as spiders were also allocated to the Control 2 (starved) group, a spider for the starved group was selected every third iteration. Spiders were kept individually throughout the course of the study.

Only female adult olive flies were fed to the spiders. Females were individually collected in 1.5 ml microtubes (Eppendorf) and stored on ice to slow down their metabolism. Each spider in the wild-type control and OX3097D-Bol treatment groups received 6 flies per week (2 flies/feeding) following the schedule in Supplementary Figure S2. Briefly, on day −7 spiders carrying an egg sac were anesthetized with 0.2 bar C0_2_, applied for less than 10 sec, and the egg sac carefully removed using tweezers. This method caused no spider mortality. Food uptake was recorded 24 hours post-feeding and uptake was measured as either; 0, 0.5, 1, 1.5 or 2 flies. Any remaining flies were removed at this point and debris was collected to prevent mold growth and further consumption. Notable behavioral changes such as withdrawal of legs under body, uncoordinated movement or curling of the legs were recorded as described elsewhere^19^. Death was recorded when the spider showed no response to prodding. At the end of the study, all spiders were killed by freezing at −20 °C for 24 h and then stored in 70% ethanol at room temperature.

The study was subjected to the validity criteria described previously^19^ and was deemed acceptable if a mortality rate of ≤6.7% in the wild-type fed control group over two weeks feeding experiment was not exceeded. Mortality rates over 6.7% in the wild-type control group would render the experiment invalid.

### Rove beetle *A*. *bilineata* rearing

The study was conducted at the SynTech Research laboratories (SynTech Research France SAS, 613 route du Bois de Loyse, 71570 La Chapelle de Guinchay, France). The non-target arthropods used were adults of *A*. *bilineata*. Beetles were reared at 20 °C ± 2 °C, relative humidity 60–90% and 16 h:8 h light:dark cycle using onion fly pupae (*D*. *antiqua;* Diptera: Anthomyiidae) as hosts for the larvae. OX3097D-Bol or wild-type olive fly larvae or *Chironomus* species larvae (control) were used as food for the adult rove beetles. Onion fly pupae and the emerging rove beetles were reared in moist standard sandy soil (LUFA 2.1, German BBA-standard test soil; LUFA, D-67346 Speyer) at 35% (±5%) maximum water-holding capacity and remoistening once a week with distilled water. Weight of the sandy soil will be used as an indirect measure of water loss. Onion fly pupae and rove beetles were reared in plastic containers (surface 150 cm^2^, volume 750 cm^3^) containing a layer of moist standard sandy soil (minimum volume 600 cm^3^/test container). Containers were sealed with a lid with an opening covered with fine mesh nylon netting (test units). Onion fly pupae were removed from the moist soil and placed in hatching test units consisting of vessels with a sieve bottom (mesh 2 mm) placed above a second vessel. The pupae were placed onto the sieve and emerging rove beetles fell through the holes and were collected in the vessel below.

### Rove beetle study

The study included four groups:

1. Treatment 1: 20 rove beetles fed OX3097D-Bol olive fly larvae at 0.10 g larvae/test unit
2. Control 1: 20 rove beetles fed wild-type olive fly larvae at 0.10 g larvae/test unit
3. Control 2: 20 rove beetles fed *Chironomus* species larvae at 0.10 g larvae/test unit
4. Control 3: 20 rove beetles fed *Chironomus* species larvae at 0.10 g larvae/test unit in the presence of toxic reference substance ROGOR PLUS at 440 g active substance/ha.

Four replicates were set-up for each treatment group. The study was design to include 28 days exposure phase to assess mortality and 49 days hatching phase to assess fecundity. Beetles were fed 3 times per week with the genetically engineered olive fly or the toxic substance during the exposure phase. The toxic reference substance (ROGOR PLUS, Plant Remedies PVT. Ltd., Patna, India) was included to demonstrate both the susceptibility of the test organism to the substance and the sensitivity of the test system. ROGOR PLUS inhibits acetylcholinesterase, an enzyme essential for the nervous system function. The toxic substance was applied once to the moist standard sandy soil using a laboratory spraying apparatus (DeVries Spray Booth Generation III, propeller application, nozzle Teeject 8002E), calibrated to deliver the exact dose. The dose used (440 g active substance/ha) should result in a minimum reduction of 50% of the reproductive capacity of *Chironomus* species compared to the untreated control (Control 2). The test units were maintained in control conditions of 20 °C ± 2 °C, relative humidity 60–90% and 16 h:8 h light:dark cycle. Rove beetle mortality was assessed over 28 days (exposure phase). Fecundity was assessed in the next generation (following exposure and during the hatching phase) by measuring the mean number of offspring produced per beetle and calculated for each group. Fecundity effect was expressed as percent reduction of the reproductive performance (mean numbers of offspring per beetle) in the treatment group in comparison to the control group and using the following expression:

$$( \% )=100\times -[1-(\text{Rt}/\text{Rc})]$$

where Rt = reproduction (number of offspring/beetle) in the treated group, Rc = reproduction in the control group.

The test was subjected to the following validity criteria: the average number of emerging rove beetles from the onion fly pupae in the Control 2 group should not be lower than 400 out of the 6000 (26.7%) flies introduced in the test units or the toxic reference substance in the Control 3 group should not result in a reduction of reproductive capacity ≤50%. Deviation from these criteria would render the study invalid as previously described^19^. The study was conducted in accordance with the recommendations of the ESCORT workshops^35^. The method was based on the guidelines proposed by Grimm *et al*.^36^, Environmental conditions were monitored throughout the study at regular intervals. The substrate was maintained at its original water content (not more than 50% of the water content evaporated).

### Statistical tests

A Fisher’s exact test (two-tailed) was performed on the emergence of parasitized and non-parasitized olive fly larvae. Generalized linear models (Glm’s) with Poisson errors or quasi Poisson errors (where over dispersion occurs) were used to determine whether there was a significant difference between life history parameters of parasitoids reared on OX3097D-Bol olive fly compared to wild-type olive fly. A Glm with Poisson/quasi Poisson errors was required to take into account that the data is count data and therefore have errors that are not normally distributed and variance that is not constant. A Student’s *t-*test (two-tailed) was carried out on the subsample of larvae that were dissected to identify the number of parasitoid eggs per olive fly larvae. Wilcox rank sum test was used to compare *Pardosa*’s food consumption. Fisher’s exact test (two-tailed) was performed on *Pardosa* mortality rates. In the *A*. *bilineata* study, a two-tailed Student’s t-test was used for comparison of mortality and fecundity between beetles fed OX3097D-Bol olive fly and wild-type olive fly and between beetles fed *Chironomus* species in the presence of ROGOR PLUS and a diet of wild-type olive flies. P-values ≤ 0.05 were considered statistically significant.

### Data availability

Data and associated protocols described in this study are available to readers upon request from the authors (enca.martin-rendon@oxitec.com). As the study was conducted by Oxitec Ltd. in 2014, biological material is no longer available.

## Electronic supplementary material

Supplementary Material
