# Supplementary material for: Exposure to genetically engineered olive fly (Bactrocera oleae) has no negative impact on three non-target organisms
Source: Sci Rep. 2017 Sep 13;7:11478. doi: 10.1038/s41598-017-11908-4 (PMC5597591; doi:10.1038/s41598-017-11908-4)
Supplement: Supplementary file 1 — Supplementary Material [file 41598_2017_11908_MOESM1_ESM.pdf]

Exposure to genetically engineered olive fly (*Bactrocera oleae*) has no negative impact on three non-target organisms

Thea Marubbi[1], Clare Cassidy[1], Esther Miller[1], Martha Koukidou[1], Enca Martin-Rendon[1], Simon Warner[1], Augusto Loni[2], Camilla Beech[1]

1. Oxitec Ltd. 71 Innovation Drive, Abingdon, Oxfordshire, OX14 4RX, United Kingdom,
2. Department of Agriculture, Food and Environment, University of Pisa, Via del Borghetto, 56124 Pisa, Italy

Supplementary material

1. **Supplementary Figure S1. Experimental timelines of the *Pardosa* spiders study.**
2. **Supplementary Figure S2. Representative images of *Psyttalia* test cages.**

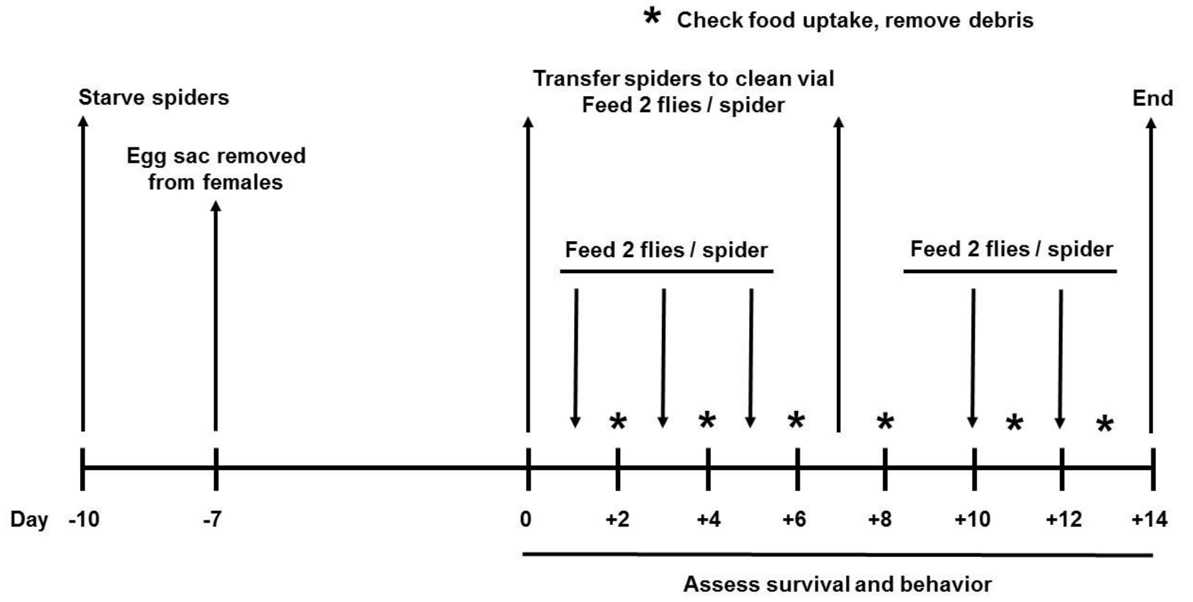

Supplementary Figure S1. Experimental timelines of the *Pardosa* spiders study. Ten days before the start of the study (day -10), all *Pardosa* spiders captured in the wild were starved. On day seven before the start of the study (day -7), the egg sac was removed from those female spiders that carry them. Spiders were transferred to a clean vial at the start of the study and again on seven days after. Spiders were given a diet of 2 flies per spider on days 0, 1, 3, 5, 7, 10 and 12. Food uptake was checked and debris removed from the vial on days 2, 4, 6, 8, 11 and 13. Survival of the spiders and changes in their behaviour were assessed daily until the end of the study on day 14.

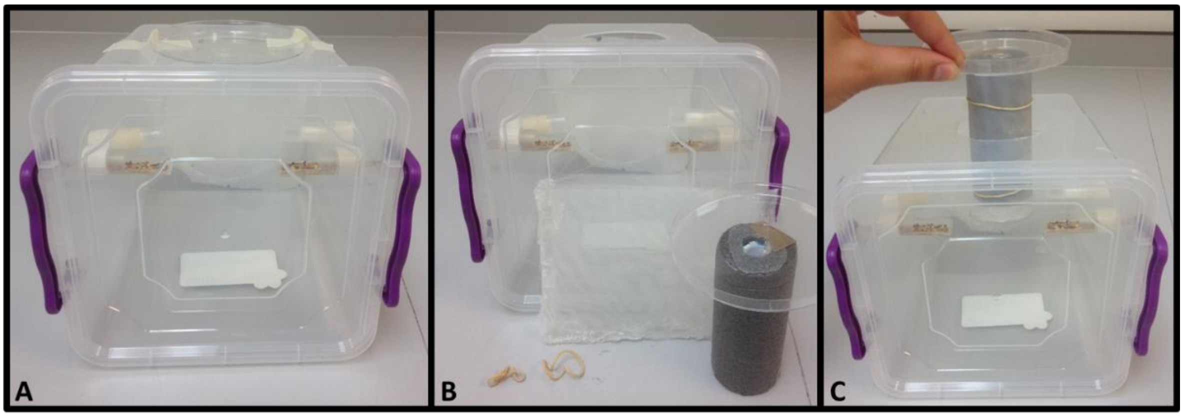

Supplementary Figure S2. Representative images of *Psyttalia* test cages. (A) Parasitoid test cage (17 cm length x 15 cm width, 15 cm height), showing food and water supply and cage hole covered by a Petri dish lid, B) Nylon mesh bag shown alongside pipe insulation, elastic bands and cage, C) Example of how to present olive fly larvae into parasitoid cage.
